# Research collaboration on community health worker programmes in low-income countries: an analysis of authorship teams and networks

**DOI:** 10.1080/16549716.2019.1606570

**Published:** 2019-05-08

**Authors:** Elma Nelisiwe Maleka, Paul Currie, Helen Schneider

**Affiliations:** a School of Public Health, University of the Western Cape, Cape Town, South Africa; b School of Public Leadership, Stellenbosch University, Stellenbosch, South Africa; c School of Public Health & SAMRC/UWC Health Services to Systems Unit, University of the Western Cape, Cape Town, South Africa

**Keywords:** Research capacity, capacity strengthening, global health partnership, low-middle-income-countries, publications

## Abstract

**Background**: Global health research partnerships, which promote the exchange of ideas, knowledge and expertise across countries, are considered key to addressing complex challenges facing health systems. Yet, many studies report inequalities in these partnerships, particularly in those between high and low-and-middle-income countries (LMICs).

**Objective**: This paper examines global research collaborations on community health worker (CHW) programmes, specifically analysing the structures of authorship teams and networks in publications reporting research on CHW programmes in low-income countries (LICs).

**Methods**: A sub-set of 206 indexed journal articles reporting on CHW programmes in LICs was purposefully selected from a prior review of research authorship on CHW programmes in all LMICs over a five year period (2012–2016). Data on country and primary organisational affiliation and number of publications for all individual authors, programme area (e.g. maternal child health) and total citations per paper were extracted and coded in excel spreadsheets. Data were then exported and analysed in Stata/ICV.14 and Gephi.

**Results**: The 206 papers were authored by 1045 authors from 299 institutions, based in 43 countries. Half (50.1%) the authors came from LIC-based institutions, 43.8% from high-income country (HIC) institutions, 2.9% from middle-income country (MIC) institutions and 3.2% had different first affiliations in different publications. Authors based in the USA (302) and UK (68) accounted for just over a third (35.4%) of all authors. Partnership patterns revealed a primary mode of North–South collaboration with authors from the US, and to a lesser extent the UK, playing central bridging roles between institutions. Strong network clusters of multiple-affiliated authors were evident in research on MCH and HIV/TB aspects of CHW programmes.

**Conclusion**: Knowledge production on CHW programmes in LICs flows predominantly through a pool of connected HIC authors and North–South collaborations. There is a need for strategies harnessing more diverse, including South–South, forms of partnership.

## Background

There has been a growth in research collaborations and partnerships addressing the health needs of low-and-middle-income countries (LMICs). As pointed out by Kenworthy et al [1] ‘the promotion of partnership with lower income countries – particularly in Africa – has become a defining feature of American global health endeavors’. Yarmoshuk et al [2] recently identified a total of 129 inter-university global health partnerships in just four African universities, most of which (60%) had been established in the prior five years.

The growth in global health partnerships and funding has enabled research on a number of health priorities in LMIC – maternal-child health [3], HIV/AIDS, malaria [4] and health policy and systems research [5]. Despite this growth in research, authorship patterns (as a reflection of power relations in research collaborations) remain unequal, with high income country authors and institutions more often than not leading research publications, especially those reporting on low-income countries [6–10].

Given this situation, there is increasing interest in examining the nature of research partnerships in global health [11,12], and related to this, the strategies and processes which enable or constrain LMIC research capacity development and South–South partnerships. For example, Crane et al [13] documented the experiences of a long term US-Uganda research collaboration in which a local NGO was established as an administrative intermediary rather than engaging directly with the local university. This was done in part because of the unwillingness of the US government to fund the local indirect costs of research. This approach to research partnerships, common in global health, acts to ‘drain rather than build capacity at African universities’ [13].

In the university partnerships reviewed by Yarmoshuk et al [2], perceived high value global health academic partnerships were those that not only supported PhD level training, but also invested in the development of teaching programmes and local infrastructure. Similarly, a long standing partnership between the Karolinska Institute in Sweden and Makerere University in Uganda not only strengthened local research capacity through a joint doctoral degree programme, but also contributed to drawing in new collaborators and funding [14]. In Bangladesh, an agreement to pool donor funding led to the establishment of the International Centre for Diarrhoeal Disease Research (ICDDR), and a better alignment of funding with research priorities [15]. Successful partnerships thus go beyond developing capacity at the level of the individual (such as post graduate training), but also seek to strengthen organisations, institutions and networks [14–19].

The rapid growth in departments of Global Health in academic institutions of the global north in the era of the Millennium Development Goals, has established North–South collaborations as the dominant mode of research engagement and partnership in LMICs. Conversely, there has been limited funding for or attention to regional or South–South partnerships [19–21]. Where these do exist, research collaborations between LMIC-based authors is often reliant on international funding, with insufficient funding for South–South partnerships being generated through local funding agencies or national governments [9]. A better understanding of the structure and dynamics of research partnership is needed to enhance collaboration among LMIC researchers and to support a long-term agenda for global health equity [8]. Such an understanding will assist in developing strategies to foster increasing participation of LMICs in collaborative learning models [9].

Conducting analyses of co-authorship networks is one way of filling knowledge gaps and can provide insight into the structure of collaborative research [22] across disciplines or geographies. Tools such as social network analysis (SNA) play an important role in understanding the functioning of such collaborations [9,23], providing an understanding of the manner in which knowledge is established and clustered, the specific relationships between researchers and between institutions, and who the key knowledge brokers may be [23].

A social *network* can be described as a group of individuals connected through some form of relationship; each participant in the network is described as an *actor* or *node*. The characteristics of these nodes, such as age or gender, are described as *attributes*. The relationships which connect these nodes are described as *lines* or *edges*, which can be either *directed* or *undirected*. For example, provision of information can flow in a directed manner from one actor to another, while a kin relationship such as between brother and sister, would be denoted as undirected, given mutual engagement in that relationship.

Social network analysis has been used to explore collaboration patterns in health research. Paula et al [9] conducted a 10-year retrospective longitudinal mapping of tuberculosis research networks in Brazil. Social network analysis proved valuable in determining ‘key central institutions maintaining network connectivity, most influential researchers that can act as advisors/experts for investment and induction policies, key researchers that could improve information exchange, systems integration and innovation within the institution, and opportunities for synergy between internal research groups working in complementary areas’ [9]. Dalglish et al [24] documented the global evolution of the integrated community case management (iCCM) strategy through the emergence of co-authorship (‘epistemic’) networks between researchers working in childhood malaria, pneumonia and diarrhoea.

Network analysis offers different kinds of insights into patterns of knowledge production and flows. Bibliometric analysis links papers or authors by citation relationships, making it useful for understanding whose knowledge outputs are attracting interest and influencing the field of study. For instance, Ramirez et al [25] examined the evolution of knowledge in the field of physical activity and public health research, mapping out the most influential publications and identifying the fundamental concepts that remain highly cited over time. On the other hand an SNA of co-authorship relationships demonstrates who participates in research [22]. In this way, while bibliometric analysis is useful for understanding how research agendas are shaped, SNA is useful for establishing how knowledge coalitions are built.

This paper examines research collaborations on community health worker (CHW) programmes, specifically analysing the structures of authorship teams and networks in publications on CHW programmes in low-income countries. Following the declaration of the Millennium Development Goals in 2000, there was a resurgence of interest in CHW programmes in LMICs, principally in response to high maternal, neonatal and under-5 mortality [10], and to the care and support needs generated by HIV [26]. These developments have been accompanied by a growth in research, with the number of publications on CHW programmes in LMICs increasing dramatically in the last decade and a half [26]. However, a recent study by the authors found that patterns of authorship on CHWs are heavily skewed towards HIC institutions, scholars and sources of funding, particularly in studies on LICs, where 60% and 69% of publications on CHW programmes in 19 LICs had a HIC lead or last author, respectively [10]. This paper extends this initial analysis by exploring in more depth the nature of research partnerships on CHWs in the sub-set of papers reporting experiences in LICs. It examines full authorship teams, institutional affiliations and networks in these publications, with the purpose of shedding light on key partnerships and collaborations, and potential leverage points for strengthening capacity in the field.

## Methods

An analysis of 206 indexed journal articles on CHW research undertaken in LICs over the five-year period of 2012 to 2016 was conducted. These publications included all LIC-based papers identified in a larger systematic review of CHW research in LMICs over the period [10]. The search terms and strategy, inclusion and exclusion criteria, and programmatic classifications adopted are detailed in two prior reviews [10,26]. In sum, the review included all English language empirical studies and reviews on CHWs obtained through EBSCOHost, specifically including MEDLINE, SocINDEX, CINAHL, PsycARTICLES, and Academic Search Complete databases. Based on the 2017 World Bank country classifications, LIC economies are those with a GNI per capita of $1,025 or less and excludes countries recently designated middle-income countries such as Zambia, Ghana and Bangladesh. Building on and extending the data extracted from the previous review, authors NM and PC independently coded data on all authors, and, where necessary, identified and merged similar author names based on discretionary criteria (if an author had two or more of the same initials, they were considered to be the same person and if they had only one initial, web searches were used to confirm or deny similarity). Three excel spreadsheets organised the attributes related to specific papers, authors, and institutions, respectively. The following attributes were extracted and coded in an Excel spreadsheet.

- Programmatic focus: maternal-child health (MCH), HIV/TB, malaria, reproductive health, non-communicable diseases (NCDs), mental health, and comprehensive (if a publication reported on two or more programmes, and/or if an author published in two or more studies on different programmatic foci), and other (diseases such bilharzia, trachoma and river blindness; or if a general health systems focus). As detailed elsewhere [10], where specific diseases (such as malaria and HIV) were studied as part of a broader child health strategy (such as iCCM), these were classified under MCH. Because community based distribution of family planning technologies has a distinct history in CHW programmes [26], publications with a reproductive health focus were analysed separately from MCH.
- Geography: country and country income classification, following the 2017 World Bank classification, of author: LIC, lower middle income (LMIC), upper middle-income (UMIC), HIC, and *multiple* if an author linked to more than one country income classification.
- Authorship: country and organisational affiliation of all authors, number of publications per author, country and country income affiliation, times cited of lead, last and both lead/last author (total citations accrued and total citations normalized per year since article publication), and organisations with 10 or more authors. For the purpose of analysis, the country of *first* institutional affiliation was used as the author’s country, despite acknowledgement that authors may be affiliated to institutions in more than one country. Similarly, the current country affiliation is not necessarily the same at the country of origin. Thus authors originating from LIC could have a HIC first affiliation and vice versa. For international organisations with offices in a number of countries, the address of the office provided by the author was the basis for classification. For example, an author working for John Snow Incorporated (JSI) in Ethiopia was classified as Ethiopia/LIC-affiliated, whereas an author based in the US office of JSI would be classified as US/HIC-affiliated. For an author who published in two or more studies and used different first affiliations, the country and organisational affiliation were recorded as multiple.
- Organisations: the affiliations of authors were coded into country level universities/research institutes; local or regional non-governmental organisations (NGO); international NGOs and global consortia/partnerships; health system entities (ministries of health or specific units/providers, if not embedded in a university/research institute); bilateral or multilateral organisations; and other (foundations, unattached consultants etc.). In the case of universities that have established international NGOs (e.g. JHPIEGO by Johns Hopkins University), head office based authors were assumed to be university associated, while affiliations to country offices were classified as international NGO.

The author spreadsheet was exported to Stata/IC V.14 and Gephi for analysis. Stata/ICV.14 was used for descriptive profile of authors, number of publications, country income affiliation, type of organisational base, and author citation time (total citations normalized per year since article publication) by country income category.

Co-authorship networks were analysed using Gephi, an open source network analysis software programme which allows visualisation and quantitative analysis of network maps, and is receptive to user-developed plugins that extend its functionality. To produce network maps of countries, authors and institutions, nodes and their attributes were imported directly into Gephi from the associated spreadsheet, while edges were imported from the same spreadsheet using line by line ‘co-occurrences’ of the journal articles, thus representing co-authorship relationships between nodes. Layout of the geographic location of publications was done using *GeoLayout*, to locate the nodes based on longitude and latitude. Final visualisation of author and institution networks for this paper was done using the *Force Atlas 1* layout algorithm to present intuitive clusters, with nodes sized by betweenness centrality and coloured by research programme (authors) and country of affiliation (institutions).

A number of common metrics were used to understand the resulting networks. These metrics characterise analysis at node, edge or network level [27]. At node level, *degree centrality* indicates the number of relationships that an actor has with those in the network, *betweenness centrality* shows ‘how much a node is located in the path between other actors,’ indicating its ability to facilitate knowledge or resource exchange, *closeness centrality* indicates how close a node is to other actors in the network or how quickly it can communicate with others, and *distance* calculates the number of edges between two nodes, indicating how many brokers may be needed for an actor to interact with another [27]. Two key characteristics of networks are *density*, which indicates the degree to which all actors in the network are connected, with implications for network cohesion, adaptability to change and innovation potential, and *centralisation* which reflects how many sub-clusters appear in the network, with implications for network power dynamics or governance, and ability to incorporate new relationships [27].

## Results

### Profile of papers and authors

The 206 indexed journal articles reported research from 19 of the 31 LICs globally, made up of 16 (out 27) LICs on the African continent, 2 (out 3) LICs in Asia, and Haiti in the Americas. The publications were authored by 1045 authors, from 299 institutions (first affiliations) based in 43 countries. There was a median of 7 authors per publication. The majority of authors (73.4%) contributed just one publication to the database, while a subset of 29 authors had 5 or more publications (Table 1).

**Table 1.** Summary characteristics of full data set, publications and authors.

	Number
Total number of publications	206
Low-income countries studied	19
Total authors	1045
*Number of countries of author affiliation*	
Total	43
Low-and-middle-income country affiliations	28
High-income country affiliations	15
*Authorship teams*	
Single-authored publications (%)	3 (1.5)
Publications with 2–5 authors (%)	34 (16.5)
Publications with ≥ 5 authors (%)	169 (82.0)
Mean (median) number of authors per publication	7.4 (7)
*Number of publications per author*	
Authors with 1 publication (%)	767 (73.4)
Authors with 2–5 publications (%)	249 (23.8)
Authors with ≥ 5 publications (%)	29 (2.8)
*Countries studied per author*	
Authors writing on 1 country (%)	1009 (96.5)
Authors writing on 2+ countries (%)	36 (3.5)

Studies undertaken in Malawi (37), Uganda (37), Ethiopia (37), Tanzania (23), Nepal (14) and Rwanda (12) together accounted for 77.6% of all publications (Table 3).

**Table 2.** Organisational affiliations of all authors (n = 1045) and in LIC with the most authors (n = 244).

	University or Research Institute (%)	International NGO or partnership (%)	Health service/ ministry (%)	Other (%)	Total (%)
All authors	587 (56.2)	229 (21.9)	122 (11.7)	107 (10.2)	1045 (100)
LIC-based	204 (38.9)	132 (25.2)	109 (20.8)	79 (15.1)	524 (100)
Other authors	383 (73.5)	97 (18.6)	13 (2.5)	28 (5.4)	521 (100)
Uganda-based	45 (50.6)	19 (21.3)	8 (9.0)	17 (19.1)	89 (100)
Ethiopia-based	30 (37.5)	34 (42.5)	10 (12.5)	6 (7.5)	80 (100)
Malawi-based	16 (21.3)	28 (37.3)	19 (25.3)	12 (16.0)	75 (100)

**Table 3.** Distribution of publications and authorship by country income category per country of study (n = 1088*).

Country of study	Total pubs of country of study	Total authors*	LMIC authors (%)	HIC authors (%)	Authors with multiple country affiliation (%)
Malawi	37	188	84 (44.7%)	97 (51.6%)	7 (3.7%)
Uganda	37	157	91 (58.0%)	59 (37.6%)	7 (4.5%)
Ethiopia	37	149	84 (56.4%)	62 (41.6%)	3 (2.0%)
Tanzania	23	137	61(45.5%)	72 (52.6%)	4 (2.9%)
Nepal	14	89	47 (52.8%)	37 (41.6%)	5 (5.6%)
Rwanda	12	73	43 (58.9%)	27 (37.0%)	3 (4.1%)
Senegal	8	60	42 (70.0%)	18 (30.0%)	0
Haiti	5	37	11 (29.7%)	26 (70.3%)	0
Madagascar	4	30	12 (40.0%)	18 (60.0%)	0
Sierra Leone	4	31	14 (45.2%)	17 (54.8%)	0
Afghanistan	6	27	5 (18.5%)	22 (81.5%)	0
Zimbabwe	4	23	14 (60.9%)	9 (39.1%)	0
Mozambique	4	23	14 (60.9%)	9 (39.1%)	0
Niger	2	20	16 (80.0%)	4 (20.0%)	0
Guinea Bissau	2	14	3 (21.4%)	11 (78.6%)	0
Burkina Faso	4	9	4 (44.4%)	5 (55.6%)	0
Liberia	1	8	6 (75.0%)	2 (25.0%)	0
DRC	1	7	7 (100.0%)	0	0
Mali	1	6	3 (50.0%)	3 (50.0%)	0
***Total***	***206***	***1088***	***561 (51.6%)***	***498 (45.8%)***	***29 (2.7%)***

*n = 1009 + 79 = 1088 (30 authors conducted studies in 2 countries; 5 authors conducted studies in 3 countries; and 1author conducted studies in 4 countries and are allocated in total authors per each country of study)

### Authorship characteristics and networks

**Figure 1.**
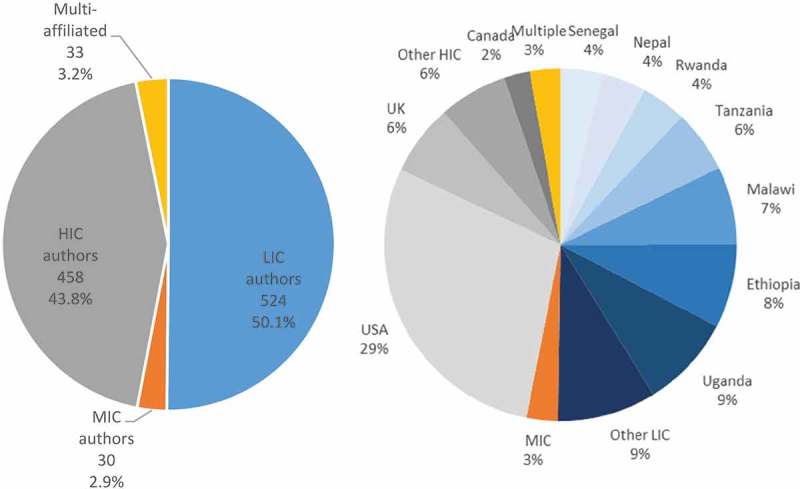
Distribution of authors by (a) country income (b) by country (n = 1045).

Authors were roughly evenly distributed between LIC (50.1%) and HIC (43.8%) affiliations, with a small proportion (2.9%) from MICs and 3.2% publishing more than one study and stating different regional first affiliations (Figure 1). Table 3 also shows the distribution of HIC vs LMIC affiliations by country of study. Authors from Uganda (89), Ethiopia (80), Malawi (75) and Tanzania (60) altogether accounted for 58.0% of LIC authors (Figure 1). Thirty authors came from eight MICs with more than half (18) based in South Africa, six from India and one each from Angola, Brazil, Kenya, Cambodia, Pakistan and Thailand. Of the HIC countries, the USA (US) had the highest number of authors (302), followed by the UK (UK) (68), Canada (25) and Netherlands (15). A total of 309 authors occupied a position of being either a lead author, last author or both, of whom 57.3% were HIC affiliated.

**Figure 5.**
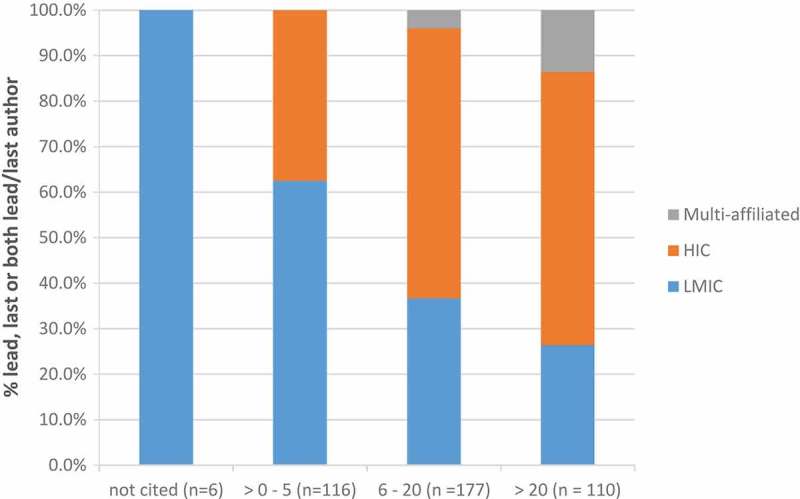
Distribution of country income class by the number of times lead or last authors (or both) cited (n = 309).

As an indicator of relative influence of these lead/last authors, Figure 5 shows the distribution of lead/last author citations by country income category. Of the 110 authors cited more than 20 times, only a quarter (26.4%) were LMIC authors.

**Figure 2.**
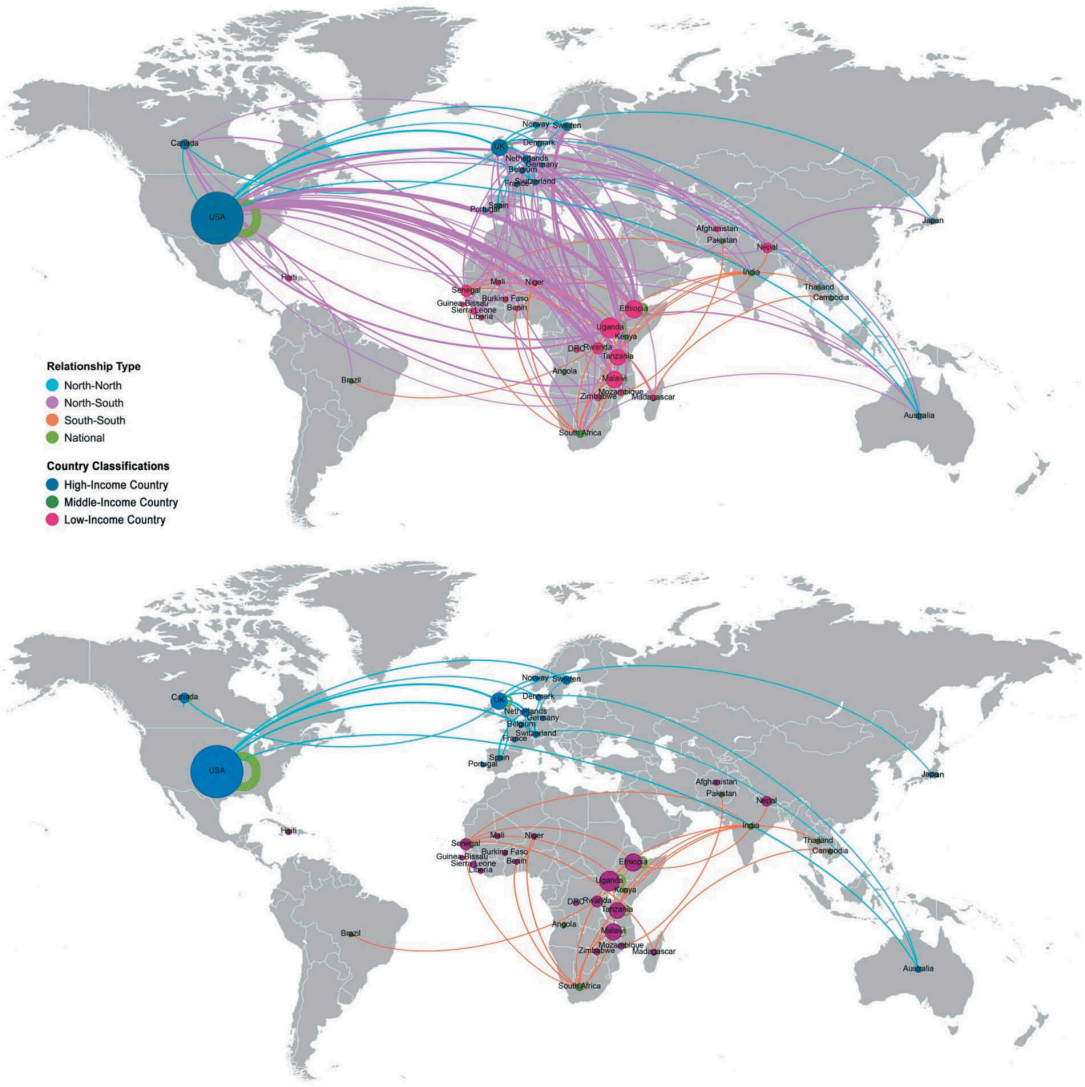
Co-authorship relationships by country; node size is number of authors affiliated in this country (given that a single paper can have multiple authors affiliated in multiple countries, papers are represented multiple times, depending on number of authors, making the nodes quantified by author X publication); the 3 single authored papers are excluded. Above shows all relationships; below shows all except north-south relationships.

Figure 2 shows the co-authorship relations between nations with geo-located nodes sized by the number of affiliated authors’ publications connected by co-authorship relationships. The dominant pattern was one of North–South co-authorship relationships, with the US as the largest single contributor to CHW publications and authors. Of the 722 global co-authorship relationships, 74 were North–North (blue lines), with the UK having the highest number of North–North relationships (42); 418 were North–South (pink lines), with US-Tanzania (37), US-Ethiopia (32) and US-Malawi (31) the most significant relationships; 33 were South–South (orange lines), with South Africa a dominant presence (15 relationships); and 197 (green lines) were co-authorship relationships within single countries, with US the largest (73) followed by Uganda (18).

**Figure 3.**
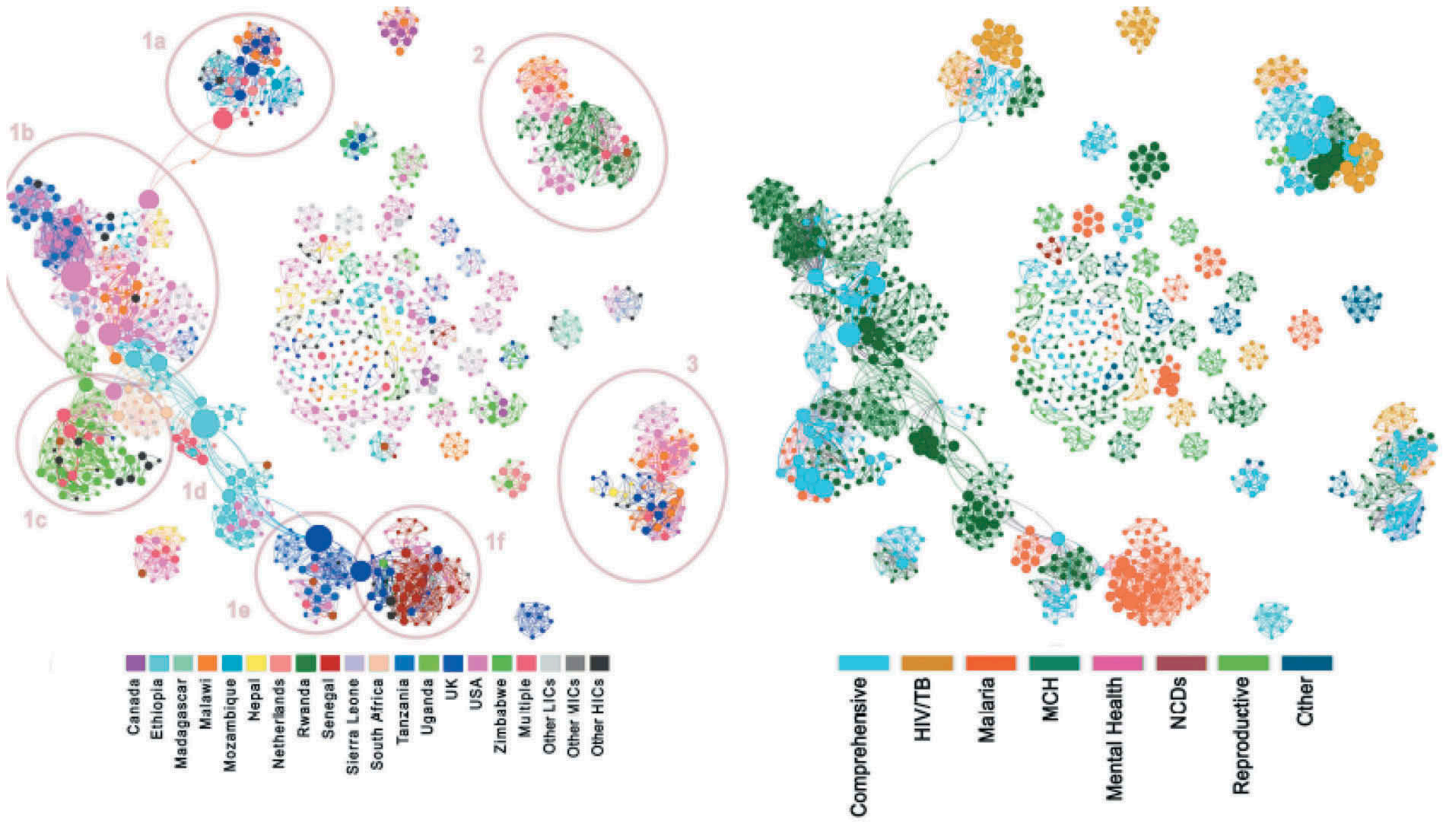
Co-author network with (a) node sized by betweenness and coloured by country of author affiliation and (b) nodes sized by citations per year and coloured by thematic area of study (n = 1045 authors).

Figure 3 shows the full co-authorship network of the 1045 authors. Figure 3(a) visualises the network nodes sized by betweenness and coloured by country of author affiliation, while Figure 3(b) has nodes sized by number of citations per year and coloured by research programme.

The first key observation was the overall fragmented nature of this network: with a density of 0.009, it is suggested that only 0.9% of all possible relationships have been made. The average co-authorship relationships between authors was 9.5 connections, ranging from a number of single authors showing no connections, to the most connected author having 50 co-authorship relationships.

The second observation was the presence of a number of clusters: one large cluster of authors (#1) spanning multiple countries and centred on maternal-child health, but linking to other programmatic areas (HIV/TB, malaria); two other smaller clusters (#2,3) and several small isolated author clusters, linked by relationships of between 1 and 3 co-authored papers. Smaller clusters were common in publications with a reproductive health theme.

In cluster 1 (Figure 3(a)), distinct groupings of countries were visible, with a number of key authors as the connectors (i.e. showing large betweenness), of researchers between country clusters. Starting with cluster 1a, a mixed group involving researchers from the UK, Netherlands, Switzerland, Ethiopia and Malawi with studies on MCH and HIV/TB (Figure 3(b)) were connected through one multi-affiliated author and a US author (bridging) to cluster 1b (Figure 3(a)), consisting of researchers from US, Tanzania, Malawi and Sierra Leone, in which a number of US based authors played strong bridging roles. From here a dense US/South Africa bridge connected to cluster 1c (Figure 3(a)), a grouping of researchers from Uganda, Denmark and Australia with a focus on MCH and Malaria (Figure 3(b)); while Ethiopian researchers, through a key UK affiliated researcher, fulfilled a bridging role (1d) to a cluster formed by Tanzania and UK researchers studying Malaria and MCH (1e). Another UK affiliated researcher acted as the main bridge to a cluster of researchers (1f) from Senegal, UK and US. Cluster 1 had a predominant focus on MCH, although with strong links to studies focusing on malaria or considering CHW programmes more comprehensively. Cluster 2 was mainly formed by researchers based in Rwanda, US and Malawi who have produced highly cited work on MCH, HIV/TB or CHW programmes more generally. Cluster 3 shows US/Malawi/UK/Nepali researchers who have produced research in multiple programmes, with foci on MCH and HIV/TB. The large number of isolated clusters display a range of research foci; notably, authors of studies on reproductive health roles of CHWs seem disproportionately disconnected (11 clusters), with a few isolated clusters of malaria (5), MCH (17) and HIV/TB (5) studies. No clusters were discerned for mental health and NCDs studies.

### Organisational characteristics and networks

The most common organisational affiliations of authors were universities or research institutes, international NGOs/consortia and ministries of health or health facilities (Table 2). There were significant differences (chi squared, p < 0001) in the organisational affiliations of LIC-based versus other (MIC, HIC and multiple) authors. Nearly three-quarters (73.5%) of HIC-based authors were affiliated to a university or research institute compared to 38.9% of LIC-based authors, who conversely were more likely to be working for an international NGO or based in the health service.

There were also variations in the distribution of organisational affiliations amongst LIC-based authors across countries (Table 2). Uganda had more than double the proportion of university affiliated authors than Malawi (50.6% vs 21.3%), while Malawi had strong representation from the Ministry of Health (25.3%) in authorship teams. Ethiopia had the highest proportion of authors working for an international NGO or partnerships (42.5%).

This profile of organisational affiliations produced the following key partnership patterns:

- North–North university/research institute partnerships, within and across HIC;
- Northern university-multilateral agency (e.g. UNICEF head office) partnerships;
- North–South university-to-university partnerships;
- Northern university–Southern ministry of health partnerships;
- Trilateral partnerships where an international NGO or consortium joined the other forms of partnership.

**Figure 4.**
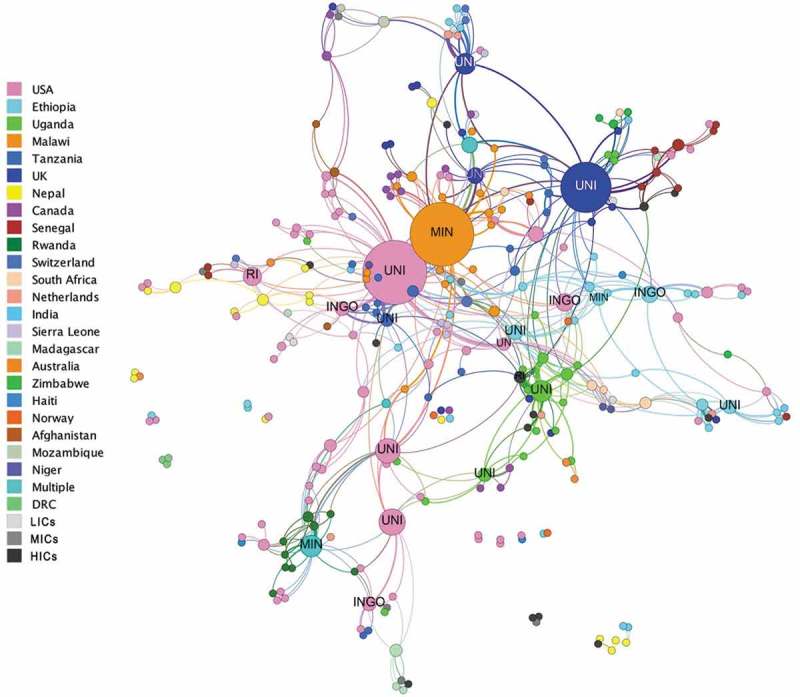
Institutional co-authorship networks – nodes sized by betweenness & coloured by country of affiliation (UNI = university; RI = research institute; UN = UN organisation; INGO = international NGO; MIN = Ministry of health).

These patterns are represented in Figure 4, which shows the institutional co-authorship network map. In this map, each node represents a specific organisation (e.g. Malawi Ministry of Health, but anonymized to a general category), coloured by the country of affiliation, and sized by the degree of *betweenness*, i.e. the extent to which the affiliated authors are bridgers other to clusters in the network. The organisational network is less modular than the author network, with a density of .016, suggesting that 1.6% of potential institutional relationships had been made. It forms a hub and spoke formation, in which US universities/research institutes fulfilled a central brokering role, along with Malawian (mostly Ministry of Health) players, to wider research hubs in Uganda, Rwanda, Tanzania and UK universities/research institutes. These central organisations produced studies in multiple programme areas (HIV, malaria, MCH), connecting to other organisations with single programmatic foci. Links between authors in LIC with similar programmatic interests were brokered by HIC organisations, mostly universities. For example, the most direct link between Ugandan and Rwandan researchers was through a US-based university; and the most direct link between Senegalese and Ugandan researchers was via a UK-based university. The thickness of the edges (lines) represents the strength of co-authorship relationships between institutions, the strongest of which was between a Ugandan university and a Northern research institute.

Table 4 lists the names of the top ranked institutions with respect to number of authors and published papers, paper citations, degree of betweenness and strength of co-authorship relationships.

**Table 4.** Organisational rankings using various metrics.

Organisations with 10 or more authors	# authors
Johns Hopkins University (incl JHPIEGO), US	75
London School of Hygiene & Tropical Medicine, UK	30
Makerere University, Uganda	28
Centers for Disease Control, US	19
Harvard University, US	14
Mbrara University of Science and Technology, Uganda	12
Ministry of Health, Malawi	12
John Snow Incorporated, Ethiopia	11
Partners in Health, Rwanda	11
Malaria Consortium, Uganda	11
Ministry of Health, Rwanda	11
University Cheikh Anta Diop, Senegal	11
University College London, UK	11
Baylor College of Medicine, US	10
Ifakara Health Institute, Tanzania	10
Muhimbili University, Tanzania	10
University of Malawi, Malawi	10
University of Zimbabwe, Zimbabwe	10
*Number of published studies*	*Ranking*
Johns Hopkins University	1
Makerere University	2
Ministry of Health, Malawi	3
London School of Hygiene and Tropical Medicine	4
Karolinska Institute, Sweden	5
*Citations*	*Ranking*
Johns Hopkins University	1
Ministry of Health, Rwanda	2
London School of Hygiene and Tropical Medicine	3
Makerere University	4
Harvard University	5
*Degree of betweenness (bridgers)*	*Ranking*
Johns Hopkins University	1
Ministry of Health, Malawi	2
London School of Hygiene and Tropical Medicine	3
University of North Carolina, US	4
Harvard University	5
Ministry of Health, Rwanda	6
Makerere University	7
Liverpool School of Tropical Medicine	8
Centers for Disease Control, US	9
Save the Children, US	10
*Strongest weighted relationships (number paper co-authors)*	*Ranking*
Makerere University & Karolinska Institute	1
Ministry of Health, Malawi & Johns Hopkins University	2
Makerere University and Malaria Consortium	3
Malaria Consortium and Karolinska Institute	4
Liverpool School of Tropical Medicine & Royal Tropical Institute (KIT), Netherlands	5

## Discussion

This paper has documented the nature of research partnerships on CHW programmes in LIC, through an analysis of co-authorship teams and networks. The analysis showed a dominant mode of North–South relationships, almost twice the number of other forms of relationship (North–North, South–South). This was confirmed by the institutional maps, in which the strongest relationships were between institutions in the North and South, with links between Southern authors typically brokered by Northern institutions.

When examining the proportional contributions of author country affiliation, the US and UK combined had around a third of authors, most of whom were affiliated to a university or research institute working in the field of global health. These and other HIC authors acted as key ‘bridgers’ in the field of CHW research and were also the most heavily cited. This reflects the growing interest in and funding of CHW initiatives associated with HIV/AIDS, TB and malaria programmes in LMICs [3,4,9].

A number of key Southern organisations did, however, play roles as hubs of authors, in particular in Uganda, which also evidenced the strongest North–South university institutional relationship in the network. Several other well-established LIC research institutions were evident in the network, even if there was little evidence of direct collaboration between them. These could form nodes of future South–South collaboration. Middle income countries with track records in CHW programme research, such as South Africa, Brazil, India and Thailand [10], are also well placed to initiate and support the development of research networks with other countries in the global south. At the same time, it is important to recognise and guard against new patterns of inequality, such as between MIC and LIC, as these emerge. As Yarmoshuk et al [2] point out ‘power dynamics exist within all partnerships: south-south partnerships should not be idealized’. Models of collaboration that enable the development of south-south-north knowledge networks, in which agendas are set collectively and learning is reciprocal, may ultimately offer the most value [28].

Ministries of health were common LIC organisational affiliations and appeared to be important focal points in the network map. The Ministries of Health of Malawi and Rwanda, in particular, were prominent, both of which have well established national CHW programmes. This positioning may have enabled LIC health system players to shape research priorities and exercise stewardship and coordination roles in research on their CHW programmes. On the other hand, this may also represent a preferred mode of research partnership of bypassing local universities/research institutions [13], where a HIC academic institution conducts research in a LIC through a partnership with the local offices of an international NGO (which may or may not be linked to the university) or directly with local health service players.

There were strong relationships between authors working on MCH and HIV/TB aspects of CHW programmes across multiple countries. Malaria research had some connections to MCH research, most likely due to the strategic uptake of integrated community case management of childhood illness (iCCM) [26]. There was a startling disconnect between authors of reproductive health studies, suggesting that more effort is needed to connect authors working in this area, many of whom are US-based, and between reproductive health and other programmatic areas. The lack of single NCD and mental health clusters suggests that these research programmes are embedded in wider research clusters as part of multiple authorships. The recent shift of CHW research focus from MCH and HIV/TB towards wider inclusion of NCDs and mental health [10] is not evidenced in the 2012–2016 sample, indicating a gap for further research and implementation.

Finally, the author network showed high modularity, which holds promise for active efforts to bridge CHW research collaborations across programmatic areas and countries. Strong authorship relationships are witnessed in the key clusters formed by researchers with common programmatic interests across HIC and LIC countries. If approached deliberately, researchers and institutions who are key bridgers could play a leading role in densifying the network, and building South–South collaborations. Funding to promote targeted author exchanges, interactions and collaborations, could see rapid changes to the structure of this network. Such funding could also strengthen the voice of key LIC ‘bridgers’ (such as Ministry or health service players) who do not traditionally occupy lead or last authorship roles. Ultimately, however, building knowledge generation capacity within LIC requires sustained investment in capacity strengthening, which considers both individuals and institutions [28,29]. This may also require changes in the way research is funded and incentives are structured in the global north [30], greater awareness of existing research capabilities within regions that can be leveraged for health research partnerships [31] and mobilisation of funding within the global south.

The field of CHW programme research is a rapidly shifting one, and the profiles and relationships portrayed represent a particular period in time, strongly influenced by the advent of the Millennium Development Goals [25]. It is possible, for example, that in an earlier era a more cohesive reproductive health research community would have been evident, or that a more current analysis would start showing the effects of new global agendas on NCDs or mental health. Similarly, research collaborations on CHW programmes should not be taken as representative of all global health research, even if they provide some window into the dynamics of this wider field [31].

A number of limitations of the analysis exist, such as the inclusion of only English language publications, which selected against research (and therefore research partnerships) amongst Francophone, Lusophone and Hispanophone countries in Africa and elsewhere. The approach to classification of publications into programme areas was based on prior knowledge and reviews of the field of CHW programme research [10]. Alternative approaches to classification (such as disaggregating MCH into component parts) may have revealed different dynamics. The widening of the sample to cover a larger timeframe could draw out how CHW programmatic foci have changed over time; a five year sample is not sufficient for observing such trends. Only first affiliations of authors were considered. A study of secondary affiliations would have provided a nuanced portrayal of the quality and duration of partnerships between HIC and LMIC researchers and institutions. Similarly, a qualitative examination of roles of different authors in publications may have provided insights into power dynamics within collaborations, and a concurrent bibliometric analysis of citation (as opposed to co-authorship) relationships would have surfaced the dominant ideas in the field. These are all potential themes for future research.

## Conclusion

Research collaboration in the field of CHW programmes in LICs revealed a strong North–South pattern with HIC authors and institutions at the core of the field. Greater diversity is required and long-term visions involving strategies to strengthen South–South collaborations are needed. South–South–North models should be explored to harness the learning across contexts, reinforce research capacity and priority setting within LMICs, and set guidelines for appropriate administration of financial support coming from HICs. This would allow all researchers to have the capacity to both set and prioritise research agendas that are locally relevant, whilst contributing to global research partnerships.

## Paper context

Inequalities in global research collaboration focusing on health priorities in LMICs still exist. Studies have provided recommendations but HIC authors remain at a core of such collaborations. This study adds to the literature by highlighting the patterns of research collaborations on CHW programmes in LICs and the unevenness of networks by country and programmatic themes. The findings point to the need for a global research collaborations that take into consideration the more equal participation of LMICs.
